# Drainage From Superior Vena Cava Improves Upper Body Oxygenation in Patients on Femoral Veno-Arterial Extracorporeal Membrane Oxygenation

**DOI:** 10.3389/fcvm.2021.807663

**Published:** 2022-02-15

**Authors:** Tong Cai, Chenglong Li, Bo Xu, Liangshan Wang, Zhongtao Du, Xing Hao, Dong Guo, Zhichen Xing, Chunjing Jiang, Meng Xin, Pengcheng Wang, Qiushi Fan, Hong Wang, Xiaotong Hou

**Affiliations:** ^1^Center for Cardiac Intensive Care, Beijing Anzhen Hospital, Capital Medical University, Beijing, China; ^2^School of Public Health, Capital Medical University, Beijing, China

**Keywords:** veno-arterial extracorporeal membrane oxygenation, differential hypoxia, upper body hypoxia, cannulation, oxygenation, cardiogenic shock

## Abstract

**Objective:**

To investigate the feasibility of drainage from the superior vena cava (SVC) to improve upper body oxygenation in patients with cardiogenic shock undergoing femoral veno-arterial extracorporeal membrane oxygenation (VA ECMO).

**Methods:**

Seventeen adult patients receiving peripheral femoral VA ECMO for circulatory support were enrolled. The femoral drainage cannula was shifted three times (from the inferior vena cava (IVC) level to the SVC level and then the IVC level again), all under ultrasound guidance, at an interval of 15 minutes. The blood gas levels of the right radial artery (RA) and SVC and cerebral oxygen saturation (ScO_2_) were measured and compared.

**Results:**

Fifteen patients (88.2%) were successfully weaned from ECMO, and 12 patients (70.6%) survived to discharge. The oxygen saturation (SO_2_) and oxygen partial pressure (PO_2_) of the RA (97.0 ± 3.5% to 98.3 ± 1.5%, *P* < 0.05, SO_2_; 127.4 ± 58.2 mmHg to 153.1 ± 67.8 mmHg, *P* < 0.05, PO_2_) and SVC (69.5 ± 9.0% to 75.7 ± 8.5%, *P* < 0.05, SO_2_; 38.5 ± 5.6 mmHg to 43.6 ± 6.4 mmHg, *P* < 0.05, PO_2_) were increased; ScO_2_ was also increased on both sides (left: 50.6 ± 8.6% to 55.0 ± 9.0%, *P* < 0.05; right: 48.7 ± 9.2% to 52.3 ± 9.8%, *P* < 0.05) when the femoral drainage cannula was shifted from the IVC level to the SVC level. When the femoral drainage cannula was shifted from SVC level to the IVC level again, the SO_2_ and PO_2_ of RA (98.3 ± 1.5% to 96.9 ± 3.2%, *P* <0.05, SO_2_; 153.1 ± 67.8 mmHg to 125.8 ± 63.3 mmHg, *P* <0.05, PO_2_) and SVC (75.7 ± 38.5% to 70.4 ± 7.6%, *P* <0.05, SO_2_; 43.6 ± 6.4 mmHg to 38.9 ± 4.5 mmHg, *P* <0.05, PO_2_) were decreased; ScO_2_ was also reduced on both sides (left: 55.0 ± 9.0% to 50.7 ± 8.2%, *P* < 0.05; right: 52.3 ± 9.8% to 48.7 ± 9.3%, *P* <0.05).

**Conclusion:**

Drainage from the SVC by shifting the cannula upward could improve upper body oxygenation in patients with cardiogenic shock undergoing femoral VA ECMO. This cannulation strategy provides an alternative solution for differential hypoxia.

## Introduction

Venous-arterial extracorporeal membrane oxygenation (VA ECMO) is a temporary mechanical circulatory support device that provides circulatory and respiratory support for patients with cardiogenic shock (1–4). However, complications, such as hemorrhage, infection, and acute kidney injury, may occur during VA ECMO support and are associated with poor outcomes (5).

Differential hypoxia is a severe complication that can lead to an insufficient oxygen supply to the heart and brain and affect the prognosis of patients (6–9). The incidence of differential hypoxia was 8.8% in patients receiving femo-femoral VA ECMO (10). Differential hypoxia occurs when femo-femoral VA ECMO patients have residual cardiac function and severely impaired pulmonary function (i.e., acute respiratory failure, redundant fluid expansion, and pulmonary edema) (11–13). In this situation, the blood to coronary arteries, cerebral blood vessels, and upper limbs is ejected from the left heart and the native lung and is poorly oxygenated. Additionally, the lower part of the body is supplied with blood from ECMO, which is well oxygenated (14, 15).

Raising the ventilator parameters and increasing ECMO flow were reported to increase oxygen delivery. However, the former may lead to lung injury (16), and the latter may cause blood cell destruction, coagulation dysfunction, and high left ventricular afterload (17–20). Differential hypoxia can also be solved by changing the position of the returning cannula, such as to the axillary artery or ascending aortic artery, or shifting to the veno-arterio-venous ECMO mode (21–25). However, additional invasive procedures are needed. A simpler alternative to relieve differential hypoxia is to place the femoral drainage cannula at the superior vena cava (SVC) level. Differential hypoxia makes blood oxygen saturations in the SVC and inferior vena cava (IVC) different. Blood in the SVC is oxygen-poor, and blood in the IVC is oxygen-rich. Therefore, drainage from the SVC makes oxygen-poor blood drain to the ECMO oxygenator, and oxygen-rich IVC blood returns to the native lung, increasing the oxygen content in the pulmonary circulation. However, that finding was only proven in animal models (26, 27).

This single-center prospective study aimed to verify the feasibility of drainage from the SVC to improve upper body oxygenation in patients with cardiogenic shock undergoing femoral VA ECMO.

## Materials and Methods

### Setting

This study was conducted between January 2021 and July 2021 at the Center for Cardiac Intensive Care, Beijing Anzhen Hospital, Capital Medical University, and was approved by the hospital's institutional review board (approved number: 2020187X). Informed consent was obtained from all the patients or their surrogates.

### Patients

This study enrolled patients (aged ≥ 18 years) with cardiogenic shock or cardiac arrest. The patients received VA ECMO for circulatory support in the intensive care unit or intraoperatively in the operating room for circulatory instability during or immediately after weaning from cardiopulmonary bypass in the primary cardiac procedure. They also received VA ECMO for refractory cardiogenic shock, malignant arrhythmia or cardiac arrest occurring in the intensive care unit (ICU), ward, or catheterization room. Patients were excluded if any one of the following was present: inability to maintain stable ECMO flow or uncontrollable surgical bleeding or bleeding at the cannulation site.

### ECMO Management

The patients had undergone ECMO implantation via peripheral cannulation through the femoral route using the cutdown method. A distal leg perfusion cannula was routinely introduced to prevent lower extremity ischemia. The ECMO flow settings were adjusted to maintain a mean arterial pressure (MAP) of 60–90 mmHg. Continuous intravenous heparin was administered to obtain an activated clotting time of 180–220 s during VA ECMO support. The 15 Fr or 17 Fr return cannula and 21 Fr femoral drainage cannula (Bio-Medicus Cannula; Medtronic Inc., Michigan, USA) were selected according to the patient's body weight. A protective lung ventilation strategy was adopted during the experiment, with a tidal volume of 6 mL/kg, a positive end-expiratory pressure of 6–8 cmH_2_O, a respiratory rate of 10–12 times/min, a FiO_2_ level of 40%, and unchanged ventilator parameters during the experiment.

### Study Protocol

**Figure 1:**
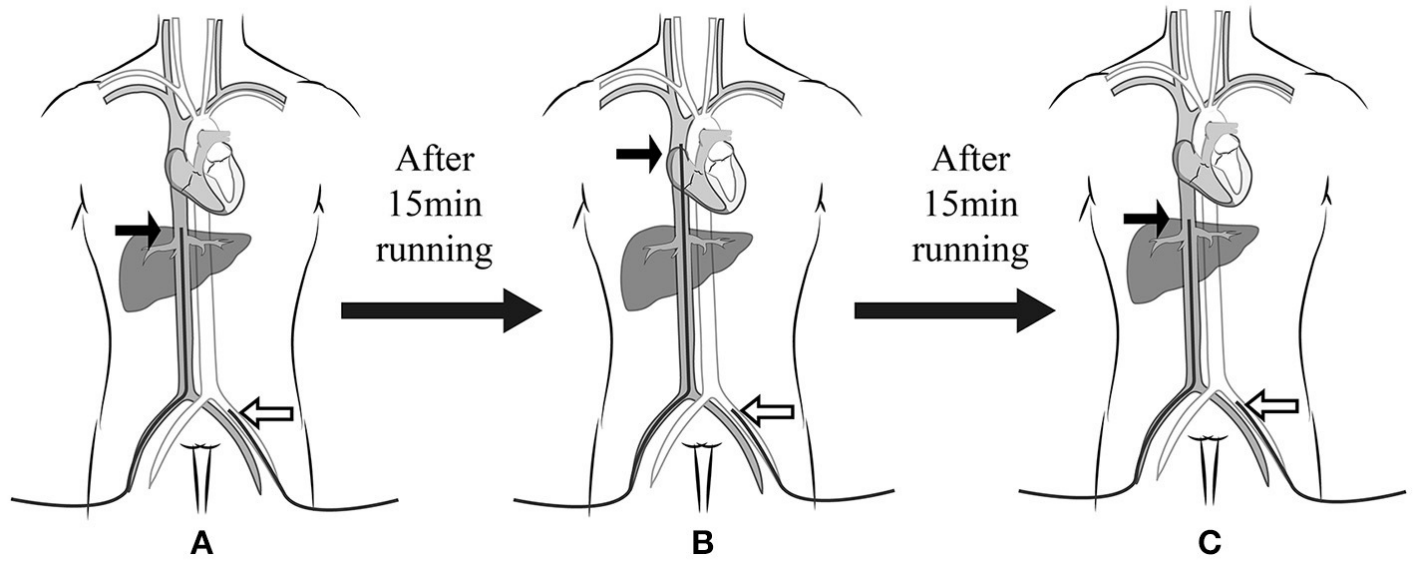
Experimental procedure. The femoral drainage cannula was shifted three times at an interval of 15 min. The femoral drainage cannula position after each move was **(A)** at the IVC level, **(B)** SVC level, and **(C)** IVC level again. The black arrow indicates the drainage cannula, and the white arrow indicates the return cannula. IVC, inferior vena cava; SVC, superior vena cava.

The experiment was performed when the cannula was adjusted after ECMO was successfully established, and the patient was provided with circulatory and respiratory support. During the experiment, the ECMO flow rate, return cannula position, drug type, and dose were kept unchanged. This experimental procedure shifted the femoral drainage cannula three times, all under ultrasound guidance (GE Vivid-i ultrasound system; GE Health care, Milwaukee, WI), at an interval of 15 min (Figure 1). The femoral drainage cannula position after each move was as follows: (1) IVC: the tip of the femoral drainage cannula was moved to the level of the opening of the hepatic vein in the IVC; (2) SVC: the tip of the femoral drainage cannula was moved to the level of the opening of the SVC; (3) IVC: the femoral drainage cannula position was the same as that in the first step. Fifteen min after each move, the blood gas levels of the right radial artery (RA) and SVC were analyzed (Stat Profile pHOx Ultra; Nova Biomedical, USA). Cerebral oxygen saturation (ScO_2_) and hemodynamic parameters (MAP, heart rate [HR], and velocity time integral [VTI] of the aortic artery) were also evaluated. Arterial and central venous catheters were placed in all the patients were placed after entering the ICU. The central venous catheter was positioned with the tip within 2 cm above the SVC. This position was verified by chest radiography and adjusted if necessary.

### Cerebral Oxygen Saturation Monitoring

ScO_2_ was measured using a tissue oxygen saturation monitor (Cerebral Oximeter Mc-2030c; CAS Medical Systems, USA). Near-infrared spectroscopy (NIRS) probes were placed on the upper edge of the patient's bilateral eyebrow arch.

### Statistical Analysis

Statistical analysis was performed using SPSS 26.0 software (SPSS Inc., Chicago, IL, USA). The data were presented as means (standard deviation) for continuous variables and numbers (percentages) for categorical variables. The differences before and after cannula shifting were analyzed using paired Student's *t*-test. A *P* value less than 0.05 was considered statistically significant.

## Results

### Patients

From January 2021 to July 2021, 17 patients had received peripheral femoral VA ECMO. The mean age was 58.2 ± 11.5 years, and 15 (88.2%) patients were male. All the patients had received circulatory support following the cardiac procedure, including coronary artery bypass grafting (CABG) (7), CABG combined with a valvular procedure (4), aortic operation (4), and a valvular procedure (2). Three patients (17.6%) had established ECMO through extracorporeal cardiopulmonary resuscitation (ECPR).

Fifteen patients (88.2%) were successfully weaned from ECMO, and 12 patients (70.6%) survived to discharge. Three patients died after ECMO weaning (2 because of multiorgan failure and 1 because of septic shock). The mean ECMO flow was 3.5 ± 0.5 L/min. The mean ICU length of stay was 14.4 ± 9.8 days. The detailed patient characteristics are shown in Table 1.

**Table 1:** Characteristics of the 17 patients.

**Variable**	**Value**
Age, mean ± SD (y)	58.2 ± 11.5
Male, *n* (%)	15(88.2%)
Comorbidity, *n* (%)	
Hypertension	11 (64.7%)
Diabetes	7 (41.2%)
Hypercholesterolemia	7 (41.2%)
LVEF on admission, mean ± SD (%)	56.4 ± 6.1
Type of surgery	
CABG	7 (41.2%)
Valve procedure	2 (11.8%)
Aortic operation	4 (23.5%)
CABG + Valve procedure	4 (23.5%)
ECMO indications, *n* (%)	
Unable to disconnect from CPB	3 (17.6%)
Cardiogenic shock	11 (64.7%)
ECMO under cardiopulmonary resuscitation, *n* (%)	3 (17.6%)
SOFA score 6h before ECMO	9.9 ± 2.0
ECMO flow, L/min	3.5 ± 0.5
ICU length of stay, d	14.4 ± 9.8
Complications, *n* (%)	
Neurological complications	1 (5.9%)
Bloodstream infection	2 (11.8%)
Bleeding	2 (11.8%)
Renal replacement therapy	6 (35.3%)
Outcomes, n (%)	
Survived to discharge	15 (88.2%)
Survived ECMO	12 (70.6%)

*LVEF, left ventricular ejection fraction; CABG, coronary artery bypass grafting; ECMO, extracorporeal membrane oxygenation; CPB, cardiopulmonary bypass; SOFA, sequential organ failure assessment; ICU, intensive care unit*.

### Hemodynamic Parameters

Seventeen data groups were collected during the study. No statistically significant differences were found in HR, MAP, or aortic VTI before and after the femoral drainage cannula was moved (*P* > 0.05) (Table 2).

**Table 2:** Hemodynamic parameters of patients.

	**HR**	***P*** **value**	**MAP**	***P*** **value**	**VTI**	***P*** **value**
IVC	95.5 ± 20.0	0.746^a^	81.1 ± 24.0	0.371^a^	12.7 ± 4.3^a^	0.284^a^
SVC	94.5 ± 20.1		78.6 ± 14.8		12.6 ± 4.4	
IVC	99.5 ± 25.3	0.090^b^	77.9 ± 14.6	0.690^b^	12.6 ± 4.5^b^	0.942^b^

*HR, heart rate; MAP, mean arterial pressure; VTI, velocity time integral*;

a *, IVC vs. SVC*;

b *, SVC vs. IVC*.

### Overall Result

**Figure 2:**
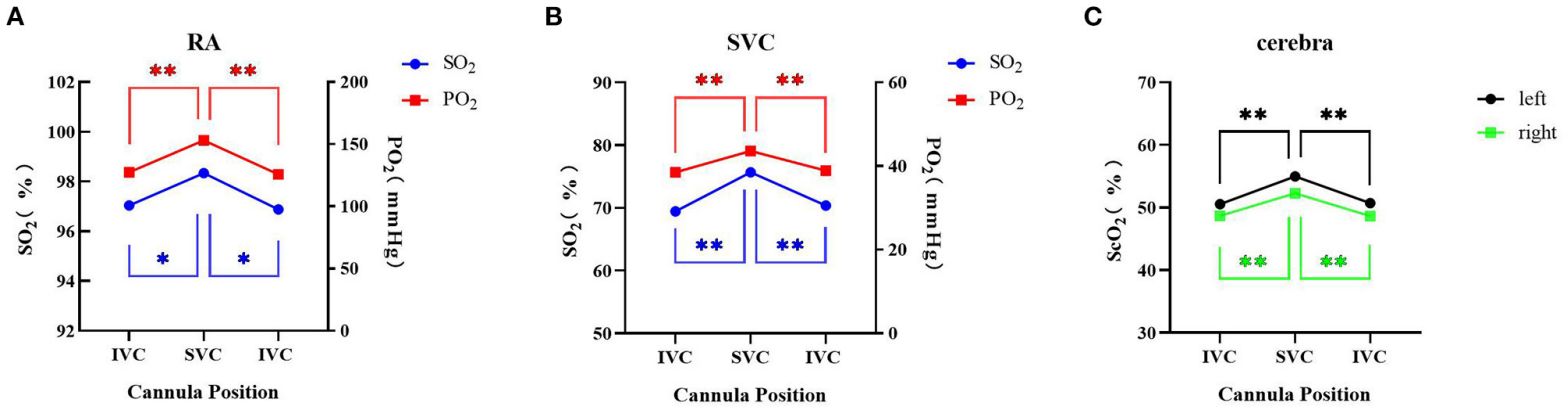
Overall blood gas analysis and cerebral oxygen saturation with different positions of the drainage cannula on femoral VA ECMO. **(A)** Right radial artery SO_2_ and PO_2_; **(B)** superior vena cava SO_2_ and PO_2_; **(C)** cerebral oxygen saturation. * Indicates *P* < 0.05 between IVC and SVC; ** Indicates *P* < 0.01 between IVC and SVC. IVC, inferior vena cava; SVC: superior vena cava; RA, right radial artery; SO_2_, oxygen saturation; PO_2_, oxygen partial pressure; ScO_2_, cerebral oxygen saturation.

Compared with the femoral drainage cannula at the IVC level, after shifting to the SVC level, the oxygen saturation (SO_2_) and oxygen partial pressure (PO_2_) of the RA (97.0 ± 3.5% to 98.3 ± 1.5%, *P* < 0.05, SO_2_; 127.4 ± 58.2 mmHg to 153.1 ± 67.8 mmHg, *P* < 0.05, PO_2_) and SVC (69.5 ± 9.0% to 75.7 ± 8.5%, *P* < 0.05, SO_2_; 38.5 ± 5.6 mmHg to 43.6 ± 6.4 mmHg, *P* < 0.05, PO_2_) were increased; ScO_2_ was also increased on both sides (left: 50.6 ± 8.6% to 55.0 ± 9.0%, *P* < 0.05; right: 48.7 ± 9.2% to 52.3 ± 9.8%, *P* < 0.05) (Figure 2).

When the femoral drainage cannula was shifted from the SVC level to the IVC level again, the SO_2_ and PO_2_ of the RA (98.3 ± 1.5% to 96.9 ± 3.2%, *P* < 0.05, SO_2_; 153.1 ± 67.8 mmHg to 125.8 ± 63.3 mmHg, *P* < 0.05, PO_2_) and SVC (75.7 ± 38.5% to 70.4 ± 7.6%, *P* < 0.05, SO_2_; 43.6 ± 6.4 mmHg to 38.9 ± 4.5 mmHg, *P* < 0.05, PO_2_) were decreased; ScO_2_ was also reduced on both sides (left: 55.0 ± 9.0% to 50.7 ± 8.2%, *P* < 0.05; right: 52.3 ± 9.8% to 48.7 ± 9.3%, *P* < 0.05) (Figure 2).

### Upper Body Hypoxia Group and Upper Body Normoxia Group

To determine whether similar changes occurred in patients with different degrees of upper body oxygen supply, we divided the patients into an upper body hypoxia group (*n* = 8) and an upper body normoxia group (*n* = 9) based on the initial central venous oxygen saturation (ScvO_2_) (measured during placement of the femoral drainage cannula on the IVC for the first time). Upper body hypoxia was defined as ScvO_2_ <70%, and normoxia was defined as ScvO_2_ ≥ 70%. The ScvO_2_ was 62.3 ± 8.2 in the upper body hypoxia group and 75.8 ± 2.5 in the upper body normoxia group (*p* < 0.01).

No significant differences were found in the hemodynamic parameters before and after the femoral drainage cannula was moved in either group (Table S1). The SO_2_ and PO_2_ of the SVC and ScO_2_ were higher with the femoral drainage cannula at the SVC level than at the IVC level in both groups (Tables 3, 4 and Figures S1, S2). However, no significant difference in the degree (SVC - IVC or SVC - IVC again) of improvement in the SO_2_ of the SVC between the groups (7.4 ± 8.5% in the upper body hypoxia group vs. 5.2 ± 4.6% in the upper body normoxia group, *P* = 0.51, SVC - IVC; 5.2 ± 5.7% in the upper body hypoxia group vs. 5.4 ± 6.6% in the upper body normoxia group, *P* = 0.963, SVC - IVC again). In the upper body hypoxia group, compared with the femoral drainage cannula at the IVC level, after shifting to the SVC level, the PO_2_ of the RA was increased, and SO_2_ showed an upward trend but no significant differences. No change was observed in the SO_2_ and PO_2_ of the RA in the upper body normoxia group (Table 3 and Figure S1).

**Table 3:** Blood gas analysis in upper body hypoxia group (ScvO2 < 70%) and upper body normoxia group (ScvO2 ≥ 70%).

		**Right radial artery**			**Superior vena cava**
	**Cannula position**	**SO_**2**_**	***P*** **value**	**PO_**2**_**	***P*** **value**	**SO_**2**_**	***P*** **value**	**PO_**2**_**	***P*** **value**
ScvO_2_ <70%	IVC	96.7 ± 3.7	0.202^a^	124.3 ± 73.7	0.006^a^	62.3 ± 8.2	0.044^a^	34.1 ± 4.0	0.023^a^
	SVC	98.1 ± 1.5		153.5 ± 84.2		69.7 ± 7.9		39.7 ± 5.5	
	IVC	96.2 ± 3.8	0.116^b^	121.3 ± 81.2	0.015^b^	64.5 ± 5.7	0.036^b^	35.9 ± 3.3	0.011^b^
ScvO_2_ ≥ 70%	IVC	97.3 ± 3.4	0.101^a^	130.2 ± 44.8	0.104^a^	75.8 ± 2.5	0.009^a^	42.4 ± 3.6	0.011^a^
	SVC	98.5 ± 1.6		152.7 ± 54.8		81.0 ± 4.7		47.1 ± 5.1	
	IVC	97.4 ± 2.9	0.102^b^	129.8 ± 47.1	0.170^b^	75.7 ± 5.0	0.041^b^	41.7 ± 3.8	0.004^b^

*ScvO_2_, central venous oxygen saturation; SO_2_, oxygen saturation; PO_2_, partial pressure of oxygen*;

a *, IVC vs. SVC*;

b *, SVC vs. IVC*.

**Table 4:** Cerebral oxygen saturation in upper body hypoxia group (ScvO2 < 70%) and upper body normoxia group (ScvO2 ≥ 70%).

	**Cannula position**	**Left**	***P*** **value**	**Right**	***P*** **value**
ScvO_2_ <70%	IVC	50.9 ± 7.2	0.003^a^	46.0 ± 7.9	0.009^a^
	SVC	56.1 ± 9.1		51.1 ± 10.8	
	IVC	50.6 ± 7.8	0.024^b^	45.3 ± 9.3	0.043 ^b^
ScvO_2_ ≥ 70%	IVC	50.3 ± 10.0	0.030^a^	50.8 ± 10.1	0.037^a^
	SVC	54.1 ± 9.3		53.1 ± 9.4	
	IVC	50.9 ± 9.0	0.031^b^	51.3 ± 8.9	0.022^b^

*ScvO_2_, central venous oxygen saturation*;

a *, IVC vs. SVC*;

b *, SVC vs. IVC*.

## Discussion

Compared with drainage from the IVC, the present study suggested that the femoral cannula drained from the SVC achieved higher SO_2_ and PO_2_ of the right radial artery in peripheral VA ECMO, representing oxygenation of the upper body. This study is the first to validate this cannulation strategy in patients, providing an alternative solution for differential hypoxia.

**Figure 3:**
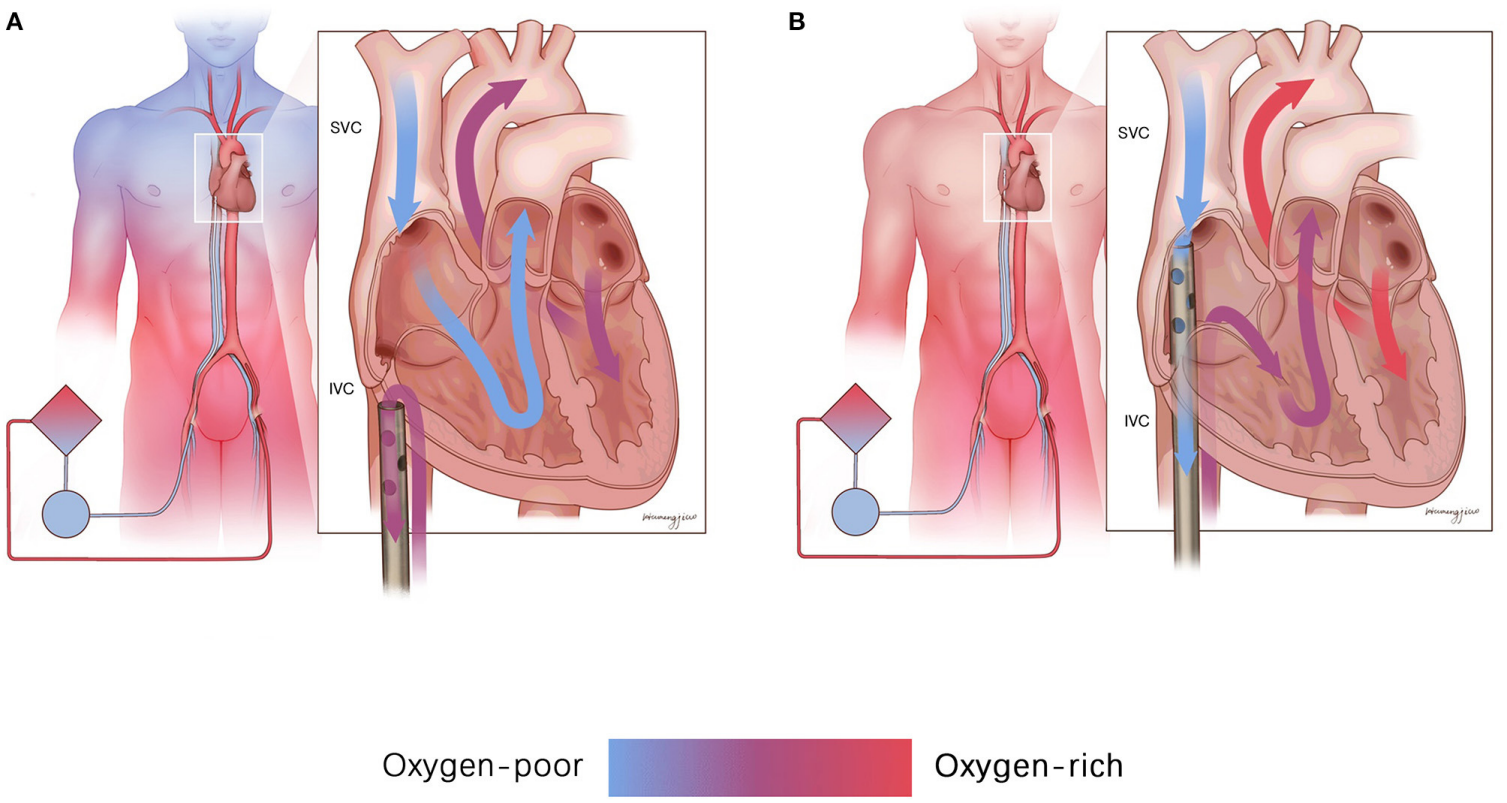
The mechanism of drainage from the SVC improves upper body oxygenation. **(A)** Drainage from the SVC made the oxygen-rich blood from the IVC drain back to the ECMO circulation, and the oxygen-poor blood from the SVC enters the right atrium and restarts the self-circulation. **(B)** Drainage from the SVC could drain out the oxygen-poor blood from the SVC into the ECMO circulation, and the oxygen-rich blood from the IVC could enter the right atrium to participate in the self-circulation. IVC, inferior vena cava; SVC, superior vena cava.

The present study found a significant increase in the SO_2_ of the RA after shifting the femoral drainage cannula position to the SVC level. We consider the mechanism as follows: a dual circulatory system with both self-circulation and ECMO circulation exists in patients with differential hypoxia. Self-circulation refers to oxygen-poor blood oxygenated by impaired lung injection through the left heart into the proximal branches of the aorta. ECMO circulation indicates well-saturated ECMO blood perfusion of the descending aorta and its distal end. Therefore, when the femoral drainage cannula is located in the IVC (Figure 3A), oxygen-rich blood returning to the IVC is drained back to the ECMO circulation, and oxygen-poor blood returning to the SVC enters the right atrium and restarts self-circulation. Therefore, the oxygen supply to the upper body worsens. Placing the femoral drainage cannula on the SVC level breaks the dual circulatory system (Figure 3B). Thus, the oxygen-poor blood from the SVC in the self-circulation is drained out by the femoral drainage cannula into the ECMO circulation, and the oxygen-rich blood from the IVC in the ECMO circulation enters the right atrium to participate in the self-circulation, eventually improving upper body oxygenation.

Lindfors et al. reached the same conclusion. They applied an artificial model to study the effect of different drainage cannula positions on upper body oxygenation in femoral VA ECMO patients. They found that the SO_2_ of RA was 64% (IVC), 75% (right atrium), and 81% (SVC) at different drainage cannula positions. They also reported a patient receiving VA ECMO using a double femoral vein drainage cannula (21 Fr, 18 and 50 cm inside the vein), and the patient developed respiratory failure with an RA SO_2_ of 57%. After moving the tip of the longer drainage cannula from the IVC to the right atrium, the SO_2_ of the RA increased by 5–10% (28).

With drainage from the SVC, upper body oxygenation could be improved. In the present study, we monitored the ScO_2_ of patients by near-infrared spectroscopy (NIRS), which was not available in the previous study. NIRS can monitor cerebral oxygen saturation, reflecting the microcirculation and tissue perfusion of patients (29). We found that placing the femoral drainage cannula on the SVC level increased ScO_2_ in femoral VA ECMO patients. The occurrence of neurological complications may be reduced theoretically. Previous experiments have also demonstrated that the animal model with differential hypoxia after receiving femoral VA ECMO shows reduced ScO_2_ (6). Choi (7) and Loftsgard (8) respectively reported a case of patients who developed differential hypoxia several days after receiving VA ECMO and developed neurological complications immediately thereafter. One of the patients presented with generalized tonic-clonic seizure, and an electroencephalogram showed delta waves suggesting diffuse cerebral cortical dysfunction (7). The other showed acute onset of neurogenic pulmonary edema, an uncommon complication of anoxic brain injury (8). However, their neurological symptoms were relieved after improving upper body oxygenation.

Previous studies have also suggested that in patients receiving femo-femoral VA ECMO with respiratory circulatory failure, myocardial hypoxia occurred as residual cardiac function increased (30). Coronary oxygen saturation was measured using proximal aortic oxygen saturation, and coronary oxygen insufficiency has been demonstrated in patients (9). Improving hypoxia of the upper body can make the myocardial oxygen supply sufficient.

Subgroup analysis was conducted. ScvO_2_ <70% reflects an insufficient oxygen supply in the upper body (31). To investigate whether this cannulation strategy shows a better oxygenation improvement effect in patients with upper body hypoxia, we classified patients with an initial measurement of ScvO_2_ <70% as the upper body hypoxia group and those with ScvO_2_ ≥ 70% as the upper body normoxia group. However, no significant difference was found in the degree of improvement in upper body oxygenation between the groups. The reason is likely that the degree of hypoxia in the upper body group was not very obvious.

Conversely, for both groups, placing the femoral drainage cannula on the SVC level increased patient ScvO_2_. Previous studies have shown that low postoperative ScvO_2_ is associated with an increased risk of complications in high-risk surgical patients, whereas elevated ScvO_2_ significantly reduces mortality in critically ill patients (32–34). However, studies on the effect of ScvO_2_ in patients receiving VA ECMO are lacking.

## Limitations

The present study was conducted in patients without severely impaired pulmonary function undergoing peripheral VA ECMO. We did not investigate the effect of SVC drainage in patients with severe differential hypoxia. Additionally, the cannulation strategy in the present study requires shifting the drainage cannula upward. The cannula is limited in length and may not reach the SVC opening in taller patients. Thus, this approach may only work for some people.

## Conclusion

Drainage from the SVC by shifting the cannula upward improved upper body oxygenation in patients with cardiogenic shock undergoing femoral VA ECMO. This cannulation strategy provides an alternative solution for differential hypoxia. The improvement of cardiac function, cerebral function and prognosis of patients using this cannulation strategy warrants further study for confirmation.

## Data Availability Statement

The raw data supporting the conclusions of this article will be made available by the authors, without undue reservation.

## Ethics Statement

The studies involving human participants were reviewed and approved by Beijing Anzhen Hospital (affiliated to Capital Medical University)'s Institutional Ethics Committee. The patients/participants provided their written informed consent to participate in this study.

## Author Contributions

TC, CL, and QF manuscript writing. TC, BX, CL, XHo, and HW study design. TC, CL, BX, and LW data analysis. ZD, XHa, DG, CJ, ZX, MX, and PW data collection. LW, XHo, XHa, and HW manuscript revision. All authors read and approved the final manuscript.

## Funding

This study was supported by grants from the Beijing Hospitals Authority Clinical Medicine Development of Special Funding Support, Code: ZYLX202111 and Beijing Hospitals Authority Ascent Plan, Code: FDL20190601.

## Conflict of Interest

The authors declare that the research was conducted in the absence of any commercial or financial relationships that could be construed as a potential conflict of interest.

## Publisher's Note

All claims expressed in this article are solely those of the authors and do not necessarily represent those of their affiliated organizations, or those of the publisher, the editors and the reviewers. Any product that may be evaluated in this article, or claim that may be made by its manufacturer, is not guaranteed or endorsed by the publisher.

## Abbreviations

VA ECMO: Veno-arterial extracorporeal membrane oxygenation
SVC: superior vena cava
IVC: inferior vena cava
RA: right radial artery
ICU: intensive care unit
SO_2_: oxygen saturation
PO_2_: oxygen partial pressure
ScO_2_: cerebral oxygen saturation
ScvO_2_: central venous oxygen saturation
VTI: velocity time integral
MAP: mean arterial pressure
HR: heart rate.
