# Supplementary material for: Drainage From Superior Vena Cava Improves Upper Body Oxygenation in Patients on Femoral Veno-Arterial Extracorporeal Membrane Oxygenation
Source: Front Cardiovasc Med. 2022 Feb 15;8:807663. doi: 10.3389/fcvm.2021.807663 (PMC8886363; doi:10.3389/fcvm.2021.807663)
Supplement: Supplementary file 1 [file Table_1.docx]

**Table S1 Hemodynamic parameters of patients in upper body hypoxia group (ScvO_2_<70%) and upper body normoxia group (ScvO_2_≥70%)**

|  | Cannula position | HR | *P* value | MAP | *P* value | VTI | *P* value |
| --- | --- | --- | --- | --- | --- | --- | --- |
| ScvO_2_<70% | IVC | 98.1±21.2 | 0.134^a^ | 78.9±16.9 | 0.890 ^a^ | 11.6±4.2 | 0.663 ^a^ |
|  | SVC | 94.8±21.0 |  | 78.1±17.7 |  | 11.5±4.5 |  |
|  | IVC | 97.6±18.7 | 0.273^b^ | 78.6±17.8 | 0.822 ^b^ | 11.4±4.5 | 0.180 ^b^ |
| ScvO_2_≥70% | IVC | 93.2±19.8 | 0.870 ^a^ | 83.1±11.4 | 0.119 ^a^ | 13.7±4.5 | 0.251 ^a^ |
|  | SVC | 94.2±20.8 |  | 79.1±12.9 |  | 13.5±4.3 |  |
|  | IVC | 101.1±31.1 | 0.193 ^b^ | 77.2±12.1 | 0.557 ^b^ | 13.6±4.6 | 0.347 ^b^ |

ScvO_2_ central venous oxygen saturation, HR heart rate, MAP mean arterial pressure, VTI velocity time integral; a: IVC vs. SVC; b: SVC vs. IVC
