# Supplementary material for: Drainage From Superior Vena Cava Improves Upper Body Oxygenation in Patients on Femoral Veno-Arterial Extracorporeal Membrane Oxygenation
Source: Front Cardiovasc Med. 2022 Feb 15;8:807663. doi: 10.3389/fcvm.2021.807663 (PMC8886363; doi:10.3389/fcvm.2021.807663)
Supplement: Supplementary file 2 [file Data_Sheet_1.docx]

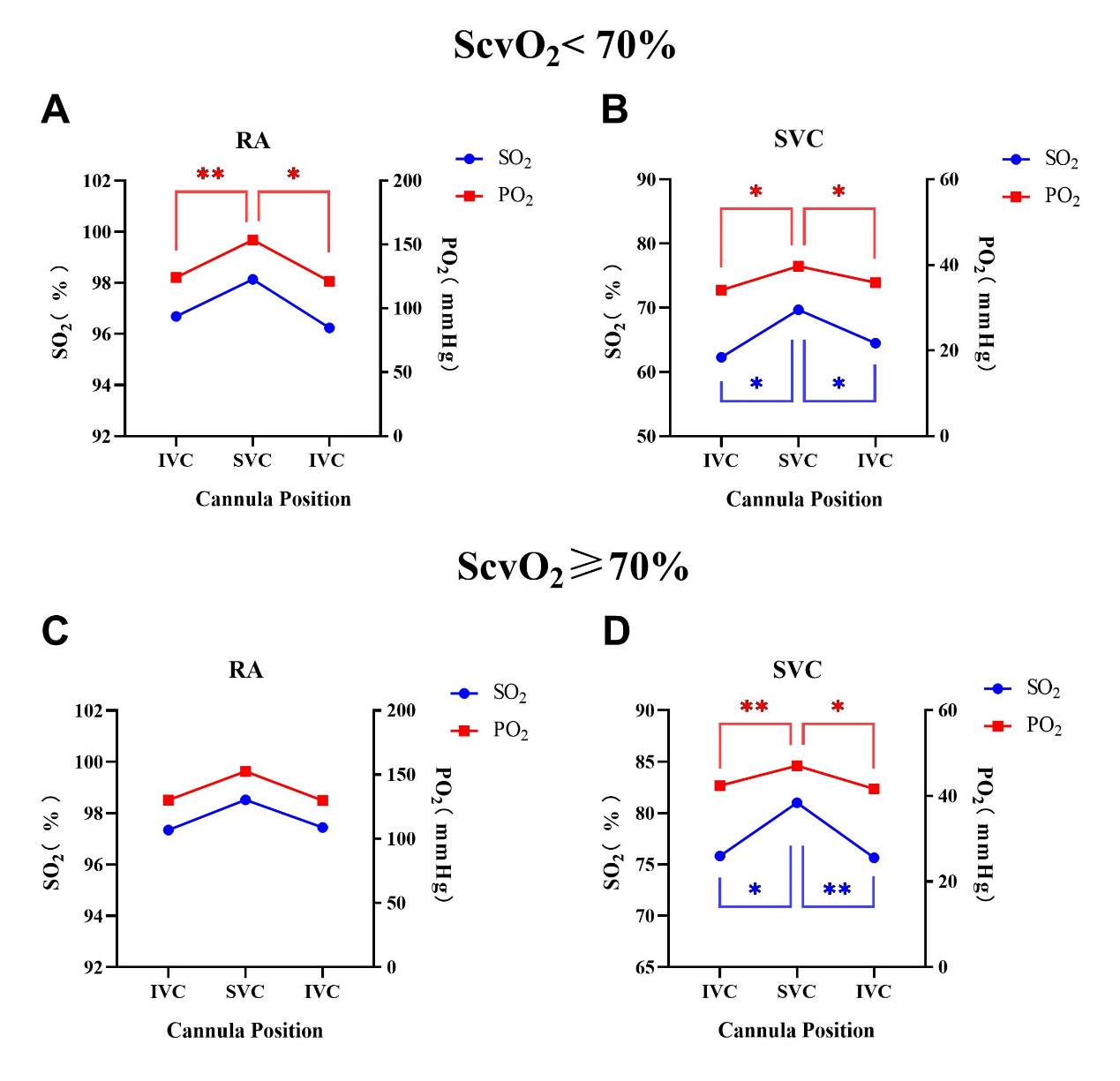


**Figure S1** SO_2_ and PO_2_ in upper body hypoxia group (ScvO_2_<70%) and upper body normoxia group (ScvO_2_≥70%) with different positions of drainage cannula on femoral VA ECMO. (A) Right radial artery SO_2_ and PO_2_ in the upper body hypoxia group; (B) Superior vena cava SO_2_ and PO_2_ in the upper body hypoxia group; (C) Right radial artery SO_2_ and PO_2_ in the upper body normoxia group; (D) Superior vena cava SO_2_ and PO_2_ in the upper body normoxia group. IVC: inferior vena cava; SVC: superior vena cava; ScvO_2_: central venous oxygen saturation; SO_2_: oxygen saturation; PO_2_: oxygen partial pressure;
