# Supplementary material for: Drainage From Superior Vena Cava Improves Upper Body Oxygenation in Patients on Femoral Veno-Arterial Extracorporeal Membrane Oxygenation
Source: Front Cardiovasc Med. 2022 Feb 15;8:807663. doi: 10.3389/fcvm.2021.807663 (PMC8886363; doi:10.3389/fcvm.2021.807663)
Supplement: Supplementary file 3 [file Data_Sheet_2.docx]

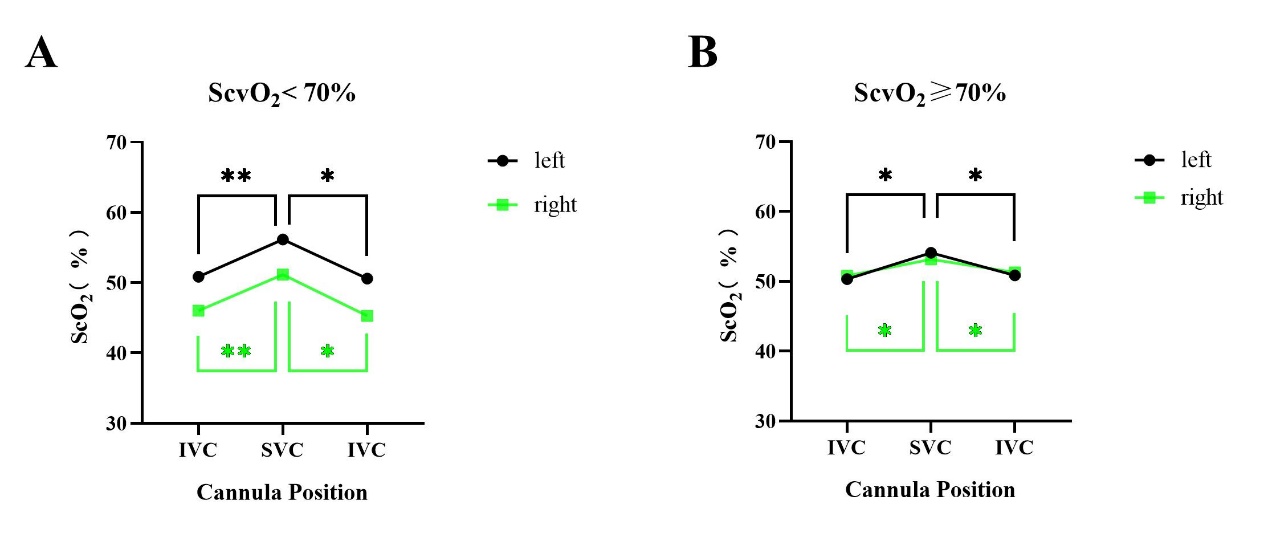


**Figure S2** Cerebral oxygen saturation in upper body hypoxia group (ScvO_2_<70%) and upper body normoxia group (ScvO_2_≥70%) with different positions of drainage cannula on femoral VA ECMO. (A) Cerebral oxygen saturation in the upper body hypoxia group; (B) Cerebral oxygen saturation in the upper body normoxia group. IVC: inferior vena cava; SVC: superior vena cava; RA: right radial artery; ScO_2_: cerebral oxygen saturation; ScvO_2_: central venous oxygen saturation
